# Effect of Autologous Platelet-Rich Plasma on Thyroidectomy Scars: A Prospective Interventional Study in a Tertiary Care Hospital in India

**DOI:** 10.7759/cureus.85404

**Published:** 2025-06-05

**Authors:** Shamila PK, Neena Chaudhary

**Affiliations:** 1 Otolaryngology - Head and Neck Surgery, Vardhman Mahavir Medical College and Safdarjung Hospital, New Delhi, IND

**Keywords:** cosmetic outcomes, platelet rich plasma, scar management, surgical wound healing, thyroidectomy

## Abstract

Introduction

Thyroid disorders are highly prevalent in India, and thyroidectomy is a frequently performed surgical procedure to manage these conditions. While conventional thyroidectomy is effective, it often results in visible neck scars, leading to patient dissatisfaction and psychosocial concerns. Platelet-rich plasma (PRP), rich in growth factors and adhesion molecules, has shown promise in enhancing wound healing and modulating scar formation. However, its efficacy in improving thyroidectomy scars remains underexplored, particularly in the Indian context.

Methods

A prospective, randomized, interventional study was conducted over 18 months (April 2023- October 2024) at the Department of Otorhinolaryngology, Vardhman Mahavir Medical College and Safdarjung Hospital, New Delhi. Thirty-two patients (aged 18-50 years) undergoing conventional thyroidectomy were randomized into two groups: Group A (control, n = 16) received standard subcuticular sutures, while Group B (intervention, n = 16) received intraoperative autologous PRP injections prior to closure. PRP was prepared by centrifuging 10 mL of the patient's blood at 2500 rpm for 10 minutes. Scar assessment was performed at 4 months postoperatively using the Patient and Observer Scar Assessment Scale (POSAS) by blinded evaluators.

Results

Baseline characteristics, including age, sex distribution, diagnosis, and type of thyroidectomy, were comparable between groups. Patient-reported outcomes indicated significantly less scar pain (mean score: 1.06 vs. 1.75; p = 0.002), reduced pigmentation difference (1.81 vs. 2.31; p = 0.032), and decreased scar thickness (1.75 vs. 2.12; p = 0.033) in the PRP group. The total POSAS patient score was significantly lower in Group B (8.81 vs. 10.56; p = 0.012), reflecting better subjective scar perception. Observer assessments corroborated these findings, noting reduced vascularity (1.06 vs. 2.31; p = 0.001), improved pigmentation (1.81 vs. 2.43; p = 0.031), and enhanced pliability (1.43 vs. 1.87; p = 0.021) in the PRP group. The total POSAS observer score favored Group B (9.12 vs. 12.18; p = 0.003).

Conclusion

Intraoperative application of autologous PRP significantly enhances scar quality following thyroidectomy, as evidenced by both patient and observer assessments. PRP offers a cost-effective, safe, and autologous approach to improve postoperative cosmetic outcomes, particularly beneficial in resource-limited settings. Further studies with larger cohorts and extended follow-up are warranted to validate these findings and establish standardized protocols for PRP application in thyroid surgery.

## Introduction

Thyroid disorders affect approximately 42 million people in India, with thyroidectomy remaining the definitive treatment for many benign and malignant conditions [1]. While conventional thyroidectomy through neck incisions is widely performed, visible scarring often leads to patient dissatisfaction, functional impairment, and psychosocial distress. Alternative approaches like transoral or robotic techniques, though scarless, remain cost-prohibitive in most Indian healthcare settings [2]. Current scar management options, including topical vitamin E, silicone sheets, and Scarguard (Scarguard Labs, Great Neck, USA), show limited efficacy in improving cosmetic outcomes [3]. 

Platelet-rich plasma (PRP) emerges as a promising biological therapy for wound healing and scar modulation. As an autologous concentrate containing growth factors (platelet-derived growth factor (PDGF), vascular endothelial growth factor (VEGF), transforming growth factor beta (TGF-β), and epidermal growth factor (EGF)) and adhesion molecules (fibrin, fibronectin), PRP accelerates tissue regeneration through angiogenesis and collagen remodeling [4]. Its established safety profile in dental and orthopedic procedures, coupled with low immunogenicity and cost-effectiveness, makes it particularly suitable for resource-constrained settings. However, its application in thyroidectomy scars remains underexplored, especially in the Indian context, where access to plastic surgeons is often limited, particularly in public healthcare settings.

The Patient and Observer Scar Assessment Scale (POSAS) provides validated metrics to evaluate scar characteristics from both patient and clinician perspectives, encompassing parameters like vascularity, pigmentation, and pliability [5]. This dual assessment is critical as patients often perceive scars differently than healthcare providers. 

Despite thyroidectomy's surgical prevalence, there exists a significant gap in accessible, effective scar prevention strategies in developing countries. PRP's biological properties suggest therapeutic potential for improving postoperative cosmesis, yet robust clinical evidence in thyroid surgery is lacking. This study aims to evaluate PRP's efficacy in thyroidectomy scar optimization using standardized POSAS metrics, addressing both a clinical need and a knowledge gap while exploring an affordable intervention for India's surgical landscape.

## Materials and methods

Study design and setting

This was a prospective, interventional, randomized comparative study conducted in the Department of Otorhinolaryngology at Vardhman Mahavir Medical College (VMMC) and Safdarjung Hospital, New Delhi, over a period of 18 months from April 2023 to October 2024. The primary objective was to compare the outcomes of thyroidectomy scars with and without the application of autologous platelet-rich plasma (PRP). A block randomization design was employed to ensure balanced allocation of participants between the two intervention groups.

Study population and sample size

The study enrolled patients aged between 18 and 50 years who required thyroidectomy for various thyroid disorders. Sample size estimation was based on findings from Kamel HH [6], who reported mean cosmesis scores of 2.26 ± 0.54 in the control group and 1.85 ± 0.13 in the PRP group. Using Snedecor and Cochran’s formula with a standard deviation (σ) of 0.39, a difference (δ) of 0.41, a significance level (α) of 5%, and power (β) of 80%, the calculated sample size was 16 participants per group, resulting in a total of 32 patients required for adequate statistical power.

Inclusion and exclusion criteria

Patients aged 18 to 50 years who were candidates for conventional thyroid surgery were included in the study. Those with comorbidities such as diabetes mellitus or hypertension, or with abnormal coagulation profiles, were excluded to minimize confounding factors and reduce surgical risk.

Methods

Eligible participants were randomized using a block randomization technique with sealed opaque envelopes to ensure allocation concealment. Blocks of 10 envelopes were prepared, with five envelopes randomly assigned to each group (Group A and Group B). Group A received standard wound closure using Vicryl subcuticular sutures, while Group B underwent PRP-augmented closure.

**Figure 1:**
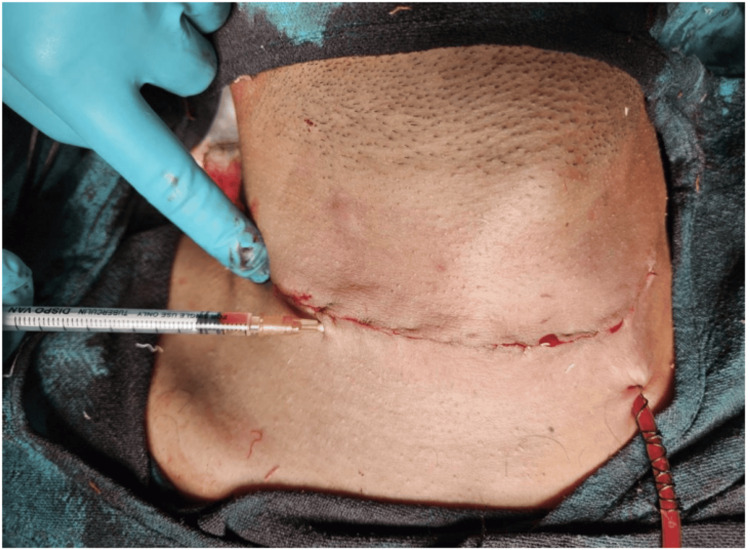
Injecting platelet-rich plasma subcutaneously along the thyroidectomy incision site

PRP was prepared intraoperatively by drawing 10 mL of autologous venous blood from the patient into a sterile citrate tube. A single-spin protocol was employed, centrifuging the sample at 2500 rpm for 10 minutes. The resulting supernatant plasma layer containing the platelet-rich fraction was carefully separated and collected. This PRP, with an estimated platelet concentration three to five times the baseline, was then injected subcutaneously along the thyroidectomy incision site (Figure 1), ensuring uniform distribution across the wound bed.

All patients received standard postoperative care, including antibiotic prophylaxis. Scar evaluation was conducted at a four-month follow-up using the Patient and Observer Scar Assessment Scale (POSAS), a validated tool for subjective and objective scar evaluation. The POSAS is a validated tool used to evaluate scar quality from both the patient's and clinician's perspectives. It includes separate scores assessing parameters like pigmentation, pliability, vascularity, and thickness, with each item rated from 1 (normal skin) to 10 (worst imaginable scar). Lower total scores indicate better scar outcomes, while higher scores reflect more severe scarring. Scars were independently assessed by both the patient and a blinded observer to reduce assessment bias.

The study protocol received ethical approval from the Institutional Ethics Committee of VMMC & Safdarjung Hospital, New Delhi (approval no. IEC/VMMC/SJH/THESIS/2023/CC-91). All participants provided written informed consent prior to enrollment, in accordance with the Declaration of Helsinki.

Statistical analysis

Data analysis was conducted using SPSS version 27 (IBM Corp., Armonk, USA). Continuous variables, including POSAS scores, were summarized as means with standard deviations and compared between groups using independent sample t-tests. Categorical variables were presented as frequencies and percentages. Graphical representations such as charts and graphs were employed to visually complement the statistical findings.

## Results

This prospective, interventional, randomized comparative study, conducted within the Department of Otorhinolaryngology at VMMC and SJH, meticulously examined the postoperative outcomes following thyroidectomy in two distinct groups of patients. A total of 32 individuals were enrolled, equally divided into Group A (the control group, n = 16) and Group B (the intervention group, n = 16). Group A received standard care, while Group B received PRP therapy. The study's design aimed to elucidate potential differences in various aspects of the postoperative experience, focusing particularly on scar-related outcomes.

The mean age in Group A was 38.81 years (SD = 10.95), while Group B had a mean age of 39.00 years (SD = 13.90). The distribution of patients by sex revealed a notable female predominance in both groups, with 93.8% females in Group A and 68.8% in Group B, resulting in an overall study population comprising 81.3% females and 18.8% males.

The primary diagnosis in both groups was predominantly solitary thyroid nodule (87.5% in Group A and 75.0% in Group B), with multinodular goiter being the other recorded diagnosis, observed in 12.5% of Group A and 25.0% of Group B. The type of thyroidectomy performed also showed a similar distribution between the groups, with hemithyroidectomy being the more frequent surgical procedure (87.5% in Group A and 75.0% in Group B) compared to total thyroidectomy (12.5% in Group A and 25.0% in Group B) (Table 1).

**Table 1:** Baseline characteristics of the study groups (n = 32)

Characteristic	Group A (n=16)	Group B (n=16)
Mean Age (Years (SD))	38.81 (10.95)	39.00 (13.90)
Sex (Female:Male)	15:1 (93.8%:6.3%)	11:5 (68.8%:31.3%)
Diagnosis (Solitary Thyroid Nodule:Multinodular Goiter)	14:2 (87.5%:12.5%)	12:4 (75.0%:25.0%)
Type of Thyroidectomy (Hemi:Total)	14:2 (87.5%:12.5%)	12:4 (75.0%:25.0%)

Further, patients in Group A reported significantly more pain over the scar (mean 1.75, SD 0.77) compared to Group B (mean 1.06, SD 0.25; p = 0.002). This statistically significant difference suggests that the intervention applied in Group A may be associated with a higher degree of postoperative pain at the surgical site as perceived by the patients themselves. However, there was no significant difference in the reported itching over the scar between the two groups (Group A mean 1.12, SD 0.34; Group B mean 1.00, SD 0.00; p = 0.154), indicating that both groups experienced similar levels of this common postoperative sensation.

Regarding scar color, patients in Group A perceived a significantly greater difference in colour compared to normal skin (mean 2.31, SD 0.70) than those in Group B (mean 1.81, SD 0.54; p = 0.032). There was no significant difference in the perceived stiffness of the scar between Group A (mean 1.56, SD 0.51) and Group B (mean 1.43, SD 0.51; p = 0.495).

Conversely, patients in Group A reported their scars as significantly thicker (mean 2.12, SD 0.50) compared to Group B (mean 1.75, SD 0.44; p = 0.033) but, no significant difference was found in the perception of scar irregularity between Group A (mean 1.68, SD 0.79) and Group B (mean 1.75, SD 0.68; p = 0.813).

The Total POSAS Patient scale score, a composite measure of the patient's overall scar assessment, was significantly higher in Group A (mean 10.56, SD 2.06) compared to Group B (mean 8.81, SD 1.60; p = 0.012), indicating a more negative overall perception of the scar among patients in the control group. Similarly, patients in Group A expressed a significantly more unfavorable overall opinion of their scar compared to normal skin (mean 2.93, SD 0.85) than those in Group B (mean 2.12, SD 0.50; p = 0.003), reinforcing the finding of a less satisfactory subjective scar experience in Group A (control group) (Table 2).

**Table 2:** Patient-reported scar outcomes (n = 32) An independent t-test was used for statistical analysis. P <0.05 was considered significant. POSAS: Patient and Observer Scar Assessment Scale

Patient-Reported Outcome	Group A (Mean ± SD)	Group B (Mean ± SD)	p-value	Significance
Pain over the scar	1.75 ± 0.77	1.06 ± 0.25	0.002	Significant
Itching over the scar	1.12 ± 0.34	1.00 ± 0.00	0.154	Not Significant
Scar color (difference from normal skin)	2.31 ± 0.70	1.81 ± 0.54	0.032	Significant
Stiffness of the scar	1.56 ± 0.51	1.43 ± 0.51	0.495	Not Significant
Scar thickness	2.12 ± 0.50	1.75 ± 0.44	0.033	Significant
Scar irregularity	1.68 ± 0.79	1.75 ± 0.68	0.813	Not Significant
Total POSAS Patient scale (higher = worse)	10.56 ± 2.06	8.81 ± 1.60	0.012	Significant
Overall opinion of the scar (higher = more unfavorable)	2.93 ± 0.85	2.12 ± 0.50	0.003	Significant

**Figure 2:**
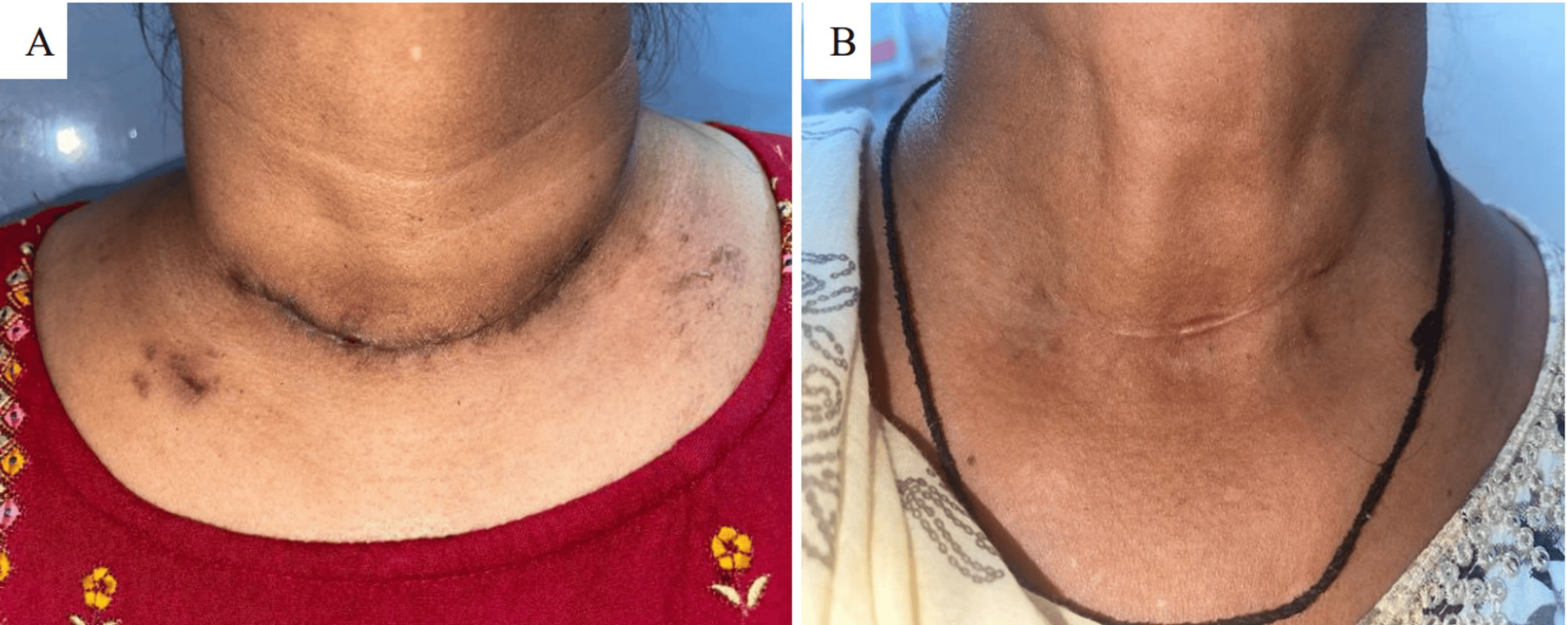
(A) Post-thyroidectomy scar on 4 months of follow-up without PRP use, and (B) post-thyroidectomy scar on 4 months of follow-up with PRP use PRP: platelet-rich plasma

In addition to the patients' self-assessments, the study incorporated an observer scale to provide an objective evaluation of the surgical scars, minimizing potential biases associated with subjective reporting. Observers noted significantly lesser vascularity in the scars of Group B patients (mean 1.06, SD 0.25; p = 0.001) compared to Group A patients (mean 2.31, SD 0.87), indicating a lower degree of redness or blood vessel prominence in the scars of the interventional group. Pigmentation differences were also significantly more pronounced in Group A (mean 2.43, SD 0.96) than in Group B (mean 1.81, SD 0.54; p = 0.031), suggesting more noticeable discoloration of the scars in the control group as perceived by the observers (Figure 2).

There was no significant difference in observed scar thickness between Group A (mean 2.25, SD 0.57) and Group B (mean 2.06, SD 0.68; p = 0.407), however, scars in Group B exhibited significantly greater relief (mean 1.50, SD 0.73; p = 0.032) compared to Group A (mean 2.06, SD 0.68).

The pliability of the scar was significantly reduced in Group A (mean 1.87, SD 0.34) compared to Group B (mean 1.43, SD 0.62; p = 0.021), indicating a less flexible and potentially more rigid scar texture in the control group upon objective palpation. No significant difference was found in the observed surface area of the scar between Group A (mean 1.25, SD 0.44) and Group B (mean 1.25, SD 0.44; p = 1.000).

The Total POSAS score, a composite measure of the observer's overall scar assessment, was significantly higher for Group A (mean 12.18, SD 2.94) compared to Group B (mean 9.12, SD 2.36; p = 0.003), indicating a less favorable overall scar appearance for Group A as judged by the observers. Finally, the observers' overall opinion of the scar was significantly more unfavorable for Group A (mean 3.06, SD 0.92) compared to Group B (mean 2.00, SD 0.36; p = 0.001), corroborating the findings from individual scar parameters and the total observer score. (Table 3)

**Table 3:** Observer-assessed scar characteristics (n = 32) An independent t-test was used for statistical analysis. P < 0.05 was considered significant. POSAS: Patient and Observer Scar Assessment Scale

Observer-Assessed Characteristic	Group A (Mean ± SD)	Group B (Mean ± SD)	p-value	Significance
Vascularity (higher = more vascular)	2.31 ± 0.87	1.06 ± 0.25	0.001	Significant
Pigmentation (higher = more difference from normal skin)	2.43 ± 0.96	1.81 ± 0.54	0.031	Significant
Thickness (higher = thicker)	2.25 ± 0.57	2.06 ± 0.68	0.407	Not Significant
Relief (higher = more raised/depressed)	2.06 ± 0.68	1.50 ± 0.73	0.032	Significant
Pliability (higher = less pliable)	1.87 ± 0.34	1.43 ± 0.62	0.021	Significant
Surface area (higher = larger)	1.25 ± 0.44	1.25 ± 0.44	1.000	Not Significant
Total POSAS Observer scale (higher = worse)	12.18 ± 2.94	9.12 ± 2.36	0.003	Significant
Overall opinion of observer (higher = more unfavorable)	3.06 ± 0.92	2.00 ± 0.36	0.001	Significant

## Discussion

Thyroid disorders and thyroidectomies are highly prevalent in India, particularly among women. Given the anterior neck location of the thyroid gland, achieving optimal functional and cosmetic outcomes is crucial for patient satisfaction. Despite the use of anti-scarring agents, their efficacy in reducing thyroidectomy scars remains limited. While advanced scarless thyroidectomy techniques exist, they require significant expertise, specialized equipment, and higher costs. As an alternative, platelet-rich plasma (PRP) - a platelet concentrate known to accelerate wound healing - has been explored for scar reduction. This study evaluated the effectiveness of PRP in 32 post-thyroidectomy cases using the Patient and Observer Scar Assessment Scale (POSAS), comparing outcomes between PRP-treated (Group B) and non-PRP-treated (Group A) patients. 

The study included a predominantly female study population (81.3%), with a mean age of 38.81 ± 10.95 years in Group A and 39.00 ± 13.90 years in Group B, showing no significant age disparity. Similar studies by Marzouki et al [7] (median age: 43 years) and Ruggiero et al [8] (median age: 46 years) reported slightly older populations. The higher proportion of females aligns with global trends, as thyroid disorders are more common in women.

In terms of diagnosis, solitary thyroid nodules (87.5% in Group A, 75% in Group B) were more frequent than multinodular goiter, contrasting with studies by Rehman et al [9] and Yoo et al [10], where multinodular goiter predominated. Most cases involved unilateral benign disease, leading to a higher incidence of hemithyroidectomy (87.5% in Group A, 75% in Group B) over total thyroidectomy. This differs from Marzouki et al's [7] findings, where total thyroidectomy was more common due to malignancy prevalence. These differences may be attributed to geographic variations in disease patterns, differences in patient selection criteria, and referral pathways. Additionally, screening practices and healthcare access may also influence the types of thyroid pathologies encountered across different populations.

PRP demonstrated significant benefits in scar-related outcomes. Patients in Group A (non-PRP) reported higher scar pain (1.75 ± 0.77 vs. 1.06 ± 0.25 in Group B, p = 0.0020), corroborating findings by Tehranian et al [11], where PRP reduced pain in cesarean scars. Pigmentation and thickness also improved significantly in the PRP group (pigmentation: 1.81 ± 0.54 vs. 2.31 ± 0.70; thickness: 1.75 ± 0.44 vs. 2.12 ± 0.50), consistent with Alser and Goutos's [12] study on acne scars. However, no significant differences were observed in itching or stiffness, contrasting with Hosseini et al's [13] findings. Overall, patients in the PRP group expressed greater satisfaction (mean score: 2.12 ± 0.50 vs. 2.93 ± 0.85), and the total POSAS patient score was lower in Group B (8.81 vs. 10.56), indicating superior scar quality. 

Observers noted significant improvements in vascularity (Group B: 1.06 ± 0.25 vs. Group A: 2.31 ± 0.87), pigmentation (1.81 ± 0.54 vs. 2.43 ± 0.96), and pliability (1.43 ± 0.62 vs. 1.87 ± 0.34) in the PRP group. These results align with Xu et al's [14] findings on PRP-enhanced neovascularization in acute wounds. While scar thickness appeared reduced in the PRP group (2.06 ± 0.68 vs. 2.25 ± 0.57), the difference was not statistically significant (p = 0.407). Surface area remained unchanged, unlike in Hosseini et al's [13] burn scar study, where PRP reduced cicatrization. The observer’s overall opinion favored the PRP group (mean score: 2.00 vs. 3.06), and the total POSAS observer score was significantly lower in Group B (9.12 vs. 12.18), reinforcing PRP’s efficacy. 

Beyond scar appearance, the PRP group exhibited faster scar maturation (~2 months), reduced drain output by postoperative day 1, and shorter hospital stays. These findings mirror Ricci et al's [15] study on parotidectomy patients, where PRP accelerated recovery.

Our study demonstrates some considerable strengths, but it is important to acknowledge the limitations as well. There is a notable difference in the proportion of females among the study groups, which could introduce gender-related bias. Additionally, important limitations include the small sample size, short follow-up period of four months, and the potential for selection bias due to the single-center design. Furthermore, the absence of platelet count measurement in the prepared PRP limits the ability to standardize or correlate platelet concentration with clinical outcomes. These factors should be considered when interpreting the generalizability of the findings.

## Conclusions

PRP application in thyroidectomy wounds significantly improved scar quality, as evidenced by lower POSAS scores in pain, pigmentation, vascularity, and overall satisfaction. While some parameters like itching and stiffness showed no significant change, the collective results highlight PRP’s potential as a cost-effective adjunct for enhancing cosmetic and functional outcomes in thyroidectomy patients. Intraoperative application of autologous PRP appears to enhance early scar quality following thyroidectomy, as reflected in both patient and observer POSAS assessments. While the findings suggest a potential role for PRP as a low-cost adjunct in cosmetic scar management, further studies with larger sample sizes, longer follow-up, and formal cost analysis are warranted to confirm its broader applicability. This study underscores PRP’s role in optimizing post-thyroidectomy recovery, particularly for cosmetically conscious patients, predominantly women, in high-prevalence regions like India.
